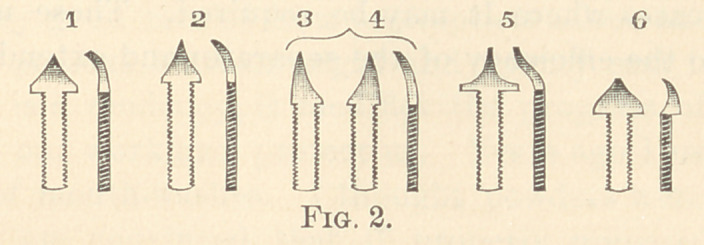# Improved Separator for the Incisors, Cuspids and Bicuspids

**Published:** 1889-02

**Authors:** W. A. Woodward

**Affiliations:** New York, N. Y.


### IMPROVED SEPARATOR FOR THE INCISORS, CUSPIDS AND BICUSPIDS.

BY W. A. WOODWARD, D.D.S., NEW YORK, N. Y.

In the construction of the separator, a description of which was published in May, 1886,* a headed screw was passed through a post set on the frame and forced between the blades or levers which separated the teeth. This headed screw, while very effective, was found to be in the way of the finishing appliances, preventing the polishing strips and disks from reaching the cervical margins of some fillings.

Fig. 1.

Two posts, A A, have since been added to the frame, E E. Through these posts pass the screws, B B, which engage the extended levers, C C, in such manner that the space between the levers is free for access in filling or finishing. These levers are operated independently of each other, which increases the adaptability of the separator. The sliding bar, D, has been much improved. It is now made flat, with threaded edges, and moves in a suitable slot in the frame, E, and is operated by turning the milled nut, F, also set in a slot at a right angle to the former. This milled nut has a number of holes drilled in it, and with any suitable pointed instrument can be turned when the separator is in position on the teeth, an advantage of much convenience where the teeth are narrow at their necks. Both the nut and the bar are securely held in place at all times and cannot become accidentally detached. The range of movement of this sliding bar, D, is considerable, which adapts the separator equally as well to broad bicuspids as to the lower incisor teeth.

Fig. 2.

There are six shapes used, and these are quickly changed by operating the milled nut, F, with the thumb and fingers. Numbers 1 and 2 are for general application. The slight variation in their

* See Dental Cosmos, vol. xxviii, page 285.

size and shape will be found effective, often where least expected. Numbers 3 and 4 are rights and lefts for the space between the cuspid and bicuspid teeth. This has proved a difficult space to manage, owing to the unequal width of the crowns of these teeth and the inclining palatal surface of the cuspid. These bars work admirably for this space, and allow the separator frame to set squarely and in line with the arch of the teeth. As the levers, C C, work independently, by moving one or the other, the frame of the separator can be made to clear or impinge upon the cusps or other surfaces of the teeth at will.

Number 5 is for short teeth in close contact, with the gum extending low down on the crowns, and the teeth projecting somewhat. It is also of use for the lower front teeth. The hold is obtained by the slender point sliding under the free margin of the gum between the teeth at their necks. Number 6 is a recent addition for bicuspids. Numbers 1 and 2 are also used for bicuspids.

In selecting a bar, one is frequently found to answer nicely for a space for which it was not intended. To illustrate: Numbers 3 and 4 can be used between a cuspid and a twisted lateral incisor, the bar for the left side sometimes answering effectively for the right.

To adjust the separator, a suitable sliding bar is first selected and placed in the frame. It is then moved, with the milled nut, until the separator will just pass over the teeth. When in position, the sliding bar is advanced between the teeth by turning the nut, D. This will bring the levers, C C, in the same space labially. The screws, B B, are then turned at intervals, moving the levers, C C, apart, thus separating the teeth. To prevent contact with the gum when the teeth are short, wedges of wood or small pieces of warmed red base plate gutta-percha may be placed between the frame of the separator and the teeth over which it passes. This will keep the levers, C C, and point of sliding bar, D, free of the gum in the few cases where it may be required. These modifications add much to the efficiency of the separator and extend its range of application.

## Figures and Tables

**Fig. 1. f1:**
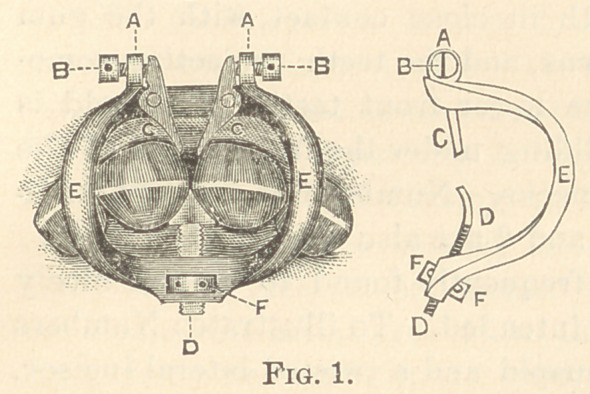


**Fig. 2. f2:**